# 6-Shogaol Protects Human Melanocytes against Oxidative Stress through Activation of the Nrf2-Antioxidant Response Element Signaling Pathway

**DOI:** 10.3390/ijms21103537

**Published:** 2020-05-16

**Authors:** Lingli Yang, Fei Yang, Lanting Teng, Ichiro Katayama

**Affiliations:** 1 Department of Pigmentation Research and Therapeutics, Graduate School of Medicine, Osaka City University, Osaka 545-0041, Japan; katayama@derma.med.osaka-u.ac.jp; 2 Department of Dermatology, Course of Integrated Medicine, Graduate School of Medicine, Osaka University, Osaka 565-0871, Japan; youhi0613@gmail.com (F.Y.); tenrantei@gmail.com (L.T.)

**Keywords:** shogaol, oxidative stress, melanocyte

## Abstract

Skin is a major target of oxidative stress. Increasing evidence suggests that oxidative stress is the cause of melanocyte disappearance in vitiligo, which is an acquired pigmentary skin disorder characterized by patches of skin that have lost pigmentation. New herbal extracts with antioxidant activity are therefore being studied. 6-Shogaol (6-SG), an active compound from ginger, is capable of attenuating oxidative stress-induced ageing and neurotoxicity. Subsequently, to investigate whether 6-SG could protect melanocytes from oxidative stress, cultured human primary epidermal melanocytes (HEMn-MPs) were treated with hydrogen peroxide (H_2_O_2_) in the presence or absence of 6-SG. The 6-SG exhibited protective effects against H_2_O_2_-induced cell death by reducing oxidative stress. In addition, the 6-SG treatment activated the Nrf2-antioxidant response element signaling pathway by upregulating the mRNA expression of the antioxidant enzyme heme oxygenase 1 (HO-1), and protein expression of Nrf2, NAD(P)H: quinine oxidoreductase 1 (Nqo1), and HO-1. Furthermore, the 6-SG also displayed protective effects on melanocytes against Rhododendrol-induced oxidative stress. We concluded that 6-SG protects melanocytes against oxidative stress in vitro, and its protective effect is associated with the activation of the Nrf2-antioxidant response element signaling pathway. 6-SG, therefore, has potential for use in the prevention of melanocyte loss in the early stages of vitiligo or other pigmentary disorders.

## 1. Introduction

Skin is the largest surface barrier organ, providing protection from harmful environmental agents such as pathogens, chemicals, and ultraviolet (UV) light. These external environmental factors directly or indirectly drive the production of various reactive oxidants, which participate in a series of physiological and pathological skin processes. Epidermal melanocytes in the skin are known to be particularly vulnerable to oxidative stress [1]. There is emerging evidence that oxidative stress can perturb the homeostasis in melanocytes and can play a significant role in the pathogenesis of vitiligo. 

Vitiligo is an acquired chronic depigmenting disease that affects 0.5–2% of the world population. It is characterized by white depigmented patches in the skin, caused by the loss of functioning melanocytes [2,3]. Vitiligo develops due to the progressive and gradual disappearance of epidermal melanocytes, which is associated with a complex interplay among biochemical, environmental, and immunological events [3,4]. Recently, oxidative stress was shown to play an important role in the development and progression of vitiligo [5,6]. In active vitiligo, high levels of oxidative stress and low levels of enzymatic and non-enzymatic antioxidants were revealed in the blood and skin from patients [7,8,9,10]. Oxidative stress has been discussed as a promising target for vitiligo drug development.

Thus, approaches and treatments using antioxidants to reduce or reverse the oxidative damage in the epidermis and even to achieve re-pigmentation are being studied. With the development of phytoextraction and medicinal chemistry technology, an increasing number of studies on organic antioxidant compounds are being carried out in order to improve the treatment of oxidative stress-associated skin diseases.

Ginger is one of the most widely known spices and a natural antioxidant [11]. It has been used as a traditional medicinal herb for thousands of years to treat many gastrointestinal, stomachic, and rheumatic disorders [12]. Recently, ginger root extract was reported to have a neuroprotective ability in β-amyloid-induced Alzheimer’s disease [13,14,15]. Due to its strong antioxidant and neuroprotective effects, ginger is considered to be a promising product for fighting the effects of aging and neurodegenerative diseases [13,14]. Melanocytes are melanin-producing cells of the skin that are derived from neural crest cells, and in a previous study, in vitro characterization of epidermal melanocyte cultures from vitiligo revealed common features with neurodegenerative diseases [16]. Ginger powder in Ayurvedic medicine [17], and ginger piece erasure in traditional Chinese medicine [18] have proven effective for the treatment of vitiligo. 

Several bioactive compounds have been identified in ginger, including 6-gingerol, 8-gingerol, 10-gingerol, and 6-Shogaol (6-SG) [12,19]. Among these ginger compounds, 6-gingerol, the most abundant bioactive compound in ginger, has been extensively studied for its various pharmacological effects including anti-inflammatory, analgesic, antipyretic, chemopreventive, and antioxidant properties [20,21,22]. Interestingly, recent studies have demonstrated that 6-SG exhibited the most potent antioxidant and anti-inflammatory properties [23,24,25]. Given the above, we focused our attention on the properties of 6-SG. The purpose of our study was to investigate whether 6-SG protects human melanocytes from oxidative stress, and to elucidate the underlying molecular mechanism of this protective effect. In the present study, we used H_2_O_2_-induced and Rhododendrol-induced oxidative stress in the normal human primary epidermal melanocytes as an in vitro model.

## 2. Results

### 2.1. 6-SG Attenuates H_2_O_2_-Induced Cytotoxicity in Human Primary Epidermal Melanocytes

First, to determine the appropriate hydrogen peroxide (H_2_O_2_) and 6-SG treatment concentrations, normal human primary epidermal melanocytes (HEMn-Mps) were exposed to different concentrations of H_2_O_2_ (Figure S1A) or 6-SG (Figure S1B) for 24 h, and cell viabilities were determined by a 3-[4–dimethylthiazol-2-yl]-2,5-diphenyltetrazoliumbromide (MTT) assay. Based on the cell viability data shown in Figure S1A and Figure S1B, H_2_O_2_ concentrations of 0.1 mM and 0.2 mM, and 6-SG concentration of 5 µM were chosen for the subsequent experiments, because 0.2 mM H_2_O_2_ significantly reduced cell viability and 5 µM 6-SG did not affect cell viability.

**Figure 1:**
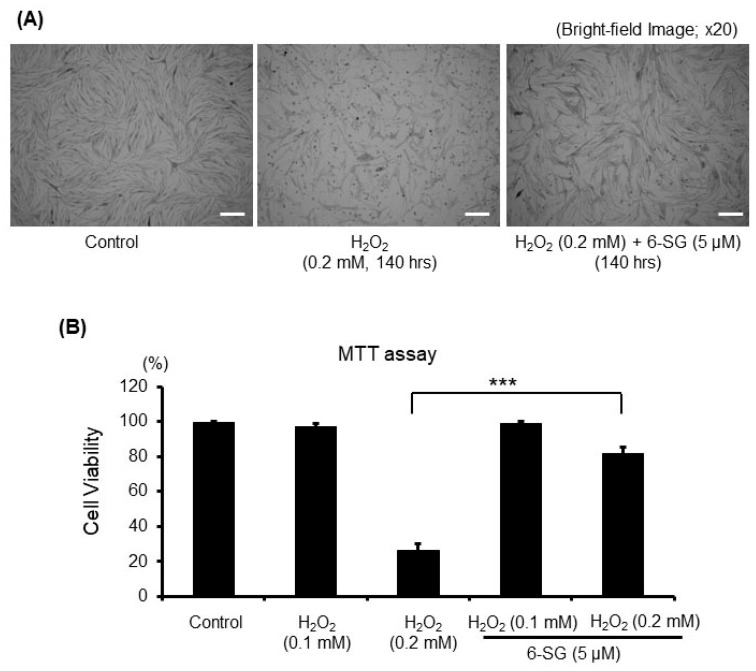
Viability of cultured human epidermal melanocytes after exposure to the indicated treatment with ^6-Shogaol (6-SG) for 140 h. (**A**) Cultured cell observation under bright-field microscopy; (**B**) Cell viability evaluation by MTT assay. White bar in (A), 50 µm. Data in (B) represent the results of three independent experiments. Data are shown as mean ± SD. ***, *p* < 0.01.

Then, to study the cyto-protective effects, HEMn-MPs were treated for 6 h with 5 µM 6-SG, and then exposed to 0.2 mM H_2_O_2_ for 140 h. Cell viability was assessed by observing cell morphology (Figure 1A) and performing MTT assays (Figure 1B). Morphological observations with light microscopy indicated that the exposure of melanocytes to 0.2 mM H_2_O_2_ for 140 h resulted in obvious membrane blebbing and cell shrinkage. In contrast, 6-SG pre-treatment attenuated these morphological changes in melanocytes (Figure 1A). In addition to the morphological evaluation, the protective effect of 6-SG against H_2_O_2_ was confirmed by the MTT assay. The viabilities of 0.2 mM H_2_O_2_-treated HEMn-MPs reduced to 26.25% for those not treated with H_2_O_2_ (Figure 1B); however, preincubation with 6-SG for 6 h reduced the cytotoxic effects of 0.2 mM H_2_O_2_ on melanocytes (Figure 1A).

### 2.2. 6-SG Attenuates Reduced Melanogenesis in H_2_O_2_-Treated Human Primary Epidermal Melanocytes

**Figure 2:**
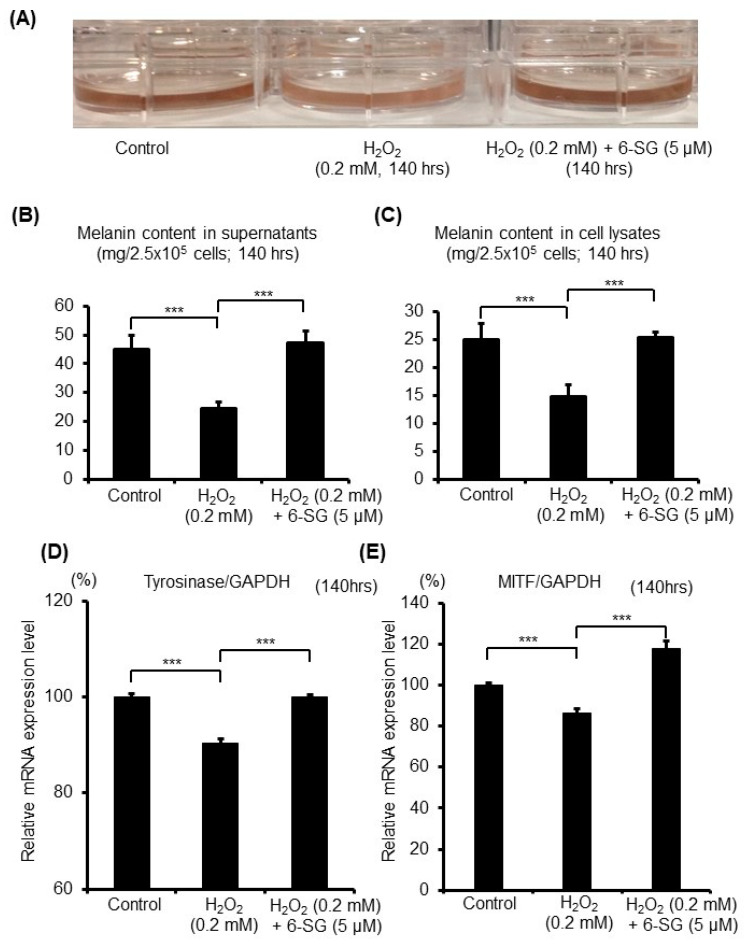
Melanin synthesis in cultured human epidermal melanocytes after 140 h exposure to the indicated treatments with 6-SG. (**A**) Photography of cultured cells with medium; (**B**) Melanin content quantification by melanin content assay in culture medium; (**C**) Melanin content quantification by melanin content assay in cell lysates; (**D**) Tyrosinase and (**E**) MITF) mRNA expression level evaluation by real-time PCR analyses with normalization to that of GAPDH. Data (B, C, D and E) represent the results of three independent experiments. Data are shown as mean ± SD. ***, *p* < 0.01.

Next, to investigate the effectiveness of 6-SG on the melanogenesis of the melanocytes that have been suffering oxidative stress, the melanin content and mRNA expression level of the key enzyme in melanogenesis were evaluated. After pretreating HEMn-MPs for 6 h with 5 µM 6-SG and exposing them to 0.2 mM H_2_O_2_ for another 140 h, the cells with 0.2 mM H_2_O_2_ were markedly less pigmented than the control cells, and 6-SG pretreatment greatly improved the medium color (Figure 2A). Through quantitative analysis, we found that the melanin content in the culture medium and cell lysates was significantly suppressed by the 0.2 mM H_2_O_2_ treatment for 140 h, compared to the control cells (Figure 2B,C). However, the cells pretreated with 6-SG significantly attenuated the H_2_O_2_-induced suppression of melanogenesis, both in the culture medium and cell lysates (Figure 2B,C).

Tyrosinase is well known as the key enzyme in melanin biosynthesis, catalyzing the only rate-limiting steps in melanogenesis [26]. After pretreating HEMn-MPs for 6 h with 5 µM 6-SG, and after exposure to 0.2 mM H_2_O_2_ for 140 h, the expression level of tyrosinase was evaluated by real-time PCR analysis (Figure 2D). The mRNA expression level of tyrosinase significantly decreased in the cells treated with 0.2 mM H_2_O_2_ compared with control cells, and 6-SG pretreatment significantly blocked the H_2_O_2_-induced decrease in tyrosinase expression (Figure 2D). Microphthalmia-associated transcription factor (MITF) is known as the main transcription factor regulating tyrosinase expression [27]. Thus, the expression level of MITF was also evaluated by real-time PCR analysis (Figure 2E). The MITF mRNA expression significantly decreased in those cells treated with 0.2 mM H_2_O_2_ compared with the control cells, and 6-SG pretreatment significantly reversed the H_2_O_2_-induced decrease in MITF expression (Figure 2E).

### 2.3. 6-SG Reduces H_2_O_2_-Induced Oxidative Stress in Human Epidermal Melanocytes

**Figure 3:**
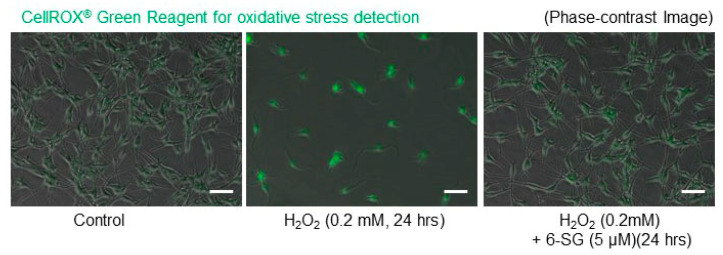
Oxidative stress in cultured human epidermal melanocytes. Oxidative stress in cultured cells was detected by CellROX^®^ Green reagent after exposure to the indicated treatments with 6-SG for 24 h. Cultured cells were photographed by phase-contrast and confocal fluorescence microscopy. Typical images obtained in three independent experiments are shown. White bar = 50 µm.

We used the cell-permeable CellROX^®^ Green Reagent to detect oxidative stress and observe the real-time visualization of intracellular oxidative stress in living melanocytes by green fluorescence. As shown in Supplementary Video S1, oxidative stress in HEMn-MPs was detected using CellROX^®^ Green reagent during exposure to 0.2 mM H_2_O_2_ for the first 12 h, where an obvious increase in green fluorescence was observed. However, as shown in Supplementary Video S2, in cells with 5 µM 6-SG pretreatment for 6 h, the H_2_O_2_-induced green fluorescence accumulation was markedly attenuated. The presentative images were taken 24 h after the indicated treatments (Figure 3). In conclusion, 6-SG preincubation markedly attenuates H_2_O_2_-induced cell death and oxidative stress in melanocytes.

### 2.4. 6-SG Activates Intrinsic Antioxidant Defense Response in Human Epidermal Melanocytes

**Figure 4:**
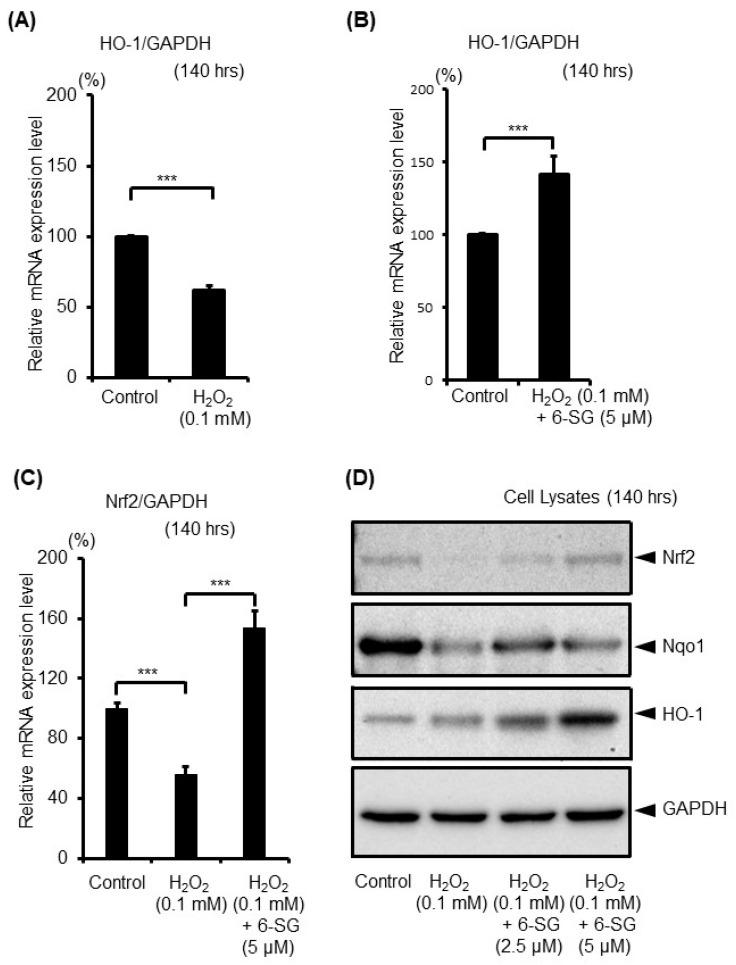
Expression level of endogenous anti-oxidant response genes in cultured human epidermal melanocytes after exposure to the indicated treatments with 6-SG for 140 h. (**A**,**B**,**C**) mRNA expression analyses using real-time PCR; (**D**) Protein expression analyses using Western blot. Data in (**A**), (**B**) and (**C**) represent the results of three independent experiments. Data in (**A**), (**B**) and (**C**) are shown as mean ± SD, normalized to GAPDH. n.s., no significant difference; ***, *p* < 0.01. Representative Western blot (of three independent experiments performed) are shown, GAPDH was used as a loading control.

6-SG is known to have its own antioxidant system. The nuclear factor E2-related factor (Nrf2)-antioxidant response element (ARE) is the most important pathway in protecting cells from oxidative stress [28,29]. It is a transcription factor that regulates the expression of antioxidant enzymes in response to oxidative stress [30]. The key downstream ARE antioxidant enzymes of Nrf2 include the heme oxygenase 1 (HO-1) and NAD(P)H: quinine oxidoreductase 1 (Nqo1) [29]. Therefore, after the 140 h treatment with 0.1 mM H_2_O_2_ with or without 6-SG pretreatment, the level of the antioxidant gene HO-1 (Figure 4A,B) and Nrf2 (Figure 4C) in melanocytes was analyzed by real-time PCR. The results demonstrated that the expression levels of HO-1 and Nrf2 were significantly downregulated in H_2_O_2_-treated cells (Figure 4A,C), and the H_2_O_2_-induced downregulation of HO-1 and Nrf2 was reversed by 6-SG pretreatment (Figure 4B,C). Following that, the expression of Nrf2 and HO-1, as well as Nqo1 protein levels, were evaluated using Western blot analyses in HEMn-MPs, after a 140 h treatment with 0.1 mM H_2_O_2_ with or without 6-SG preincubation (Figure 4D). The H_2_O_2_ treatment induced a marked decrease in Nrf2, HO-1, and Nqo1 protein expression, whereas 6-SG pretreatment reversed the H_2_O_2_-induced a decrease in these proteins (Figure 4C).

### 2.5. 6-SG Protects Melanocytes Against Rhododendrol-Induced Oxidative Stress

**Figure 5:**
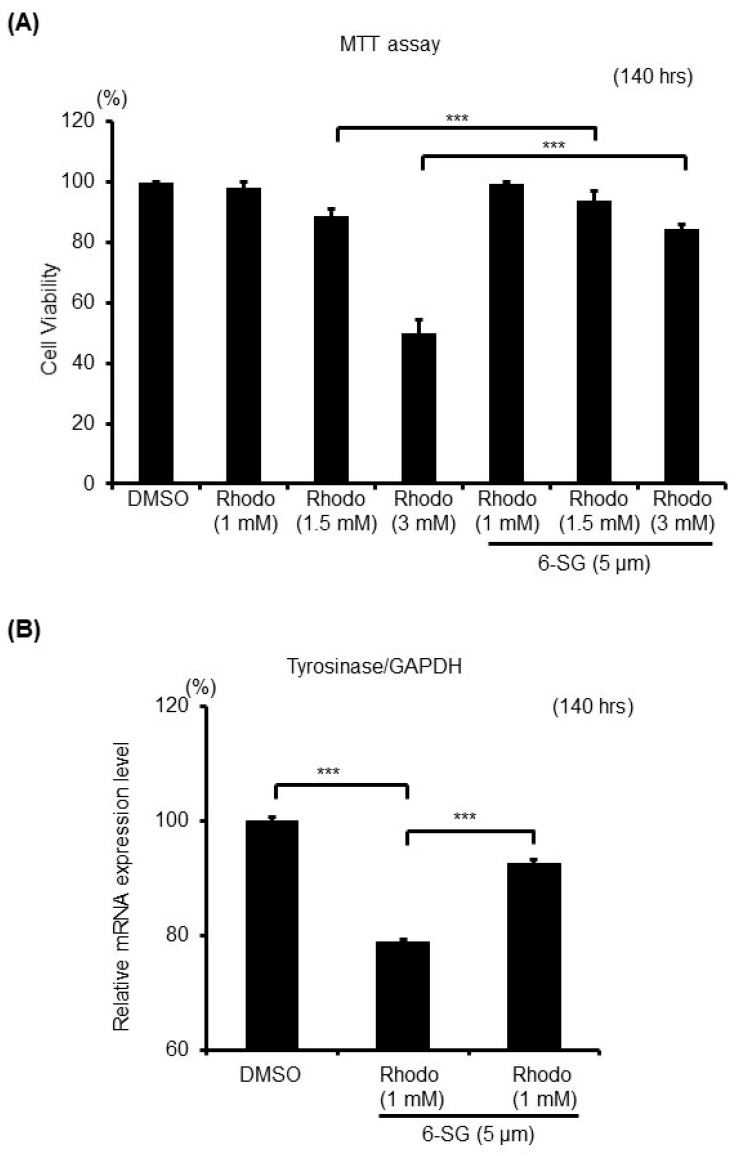
Cell viability and melanogenesis-related gene expression in cultured human epidermal melanocytes after exposure to the indicated treatment with 6-SG for 140 h. (**A**) Cell viability assessment by MTT assay; (**B**) Tyrosinase mRNA expression level analyses using real-time PCR. Data in (**A**) and (**B**) represent the results of three independent experiments. Data are shown as mean ± SD. ***, *p* < 0.01. Data in (**B**) are normalized to GAPDH.

Rhododendrol [4-(4-hydroxyphenyl)-2-butanol, Rhododenol^®^] was previously used as a skin-whitening cosmetic. These cosmetics were withdrawn from the market in 2013 after Rhododendrol reportedly caused a depigmentation disorder [31]. A previous report suggested that the toxicity of Rhododendrol towards melanocytes is caused by its tyrosinase-catalyzed oxidation and the production of cytotoxic reactive oxygen species (ROS) [32,33,34]. In the present study, we investigated whether 6-SG could protect melanocytes against Rhododendrol-induced cytotoxicity (Figure 5). After pretreating HEMn-MPs for 6 h with 5 µM 6-SG, and after subsequent exposure to 1 mM-3 mM Rhododendrol for 140 h, cell viabilities were assessed by the MTT assay (Figure 5A), and the gene expression level of tyrosinase was evaluated by real-time PCR analysis (Figure 5B). We found that 6-SG pretreatment significantly attenuated Rhododendrol-induced cell death in cultured melanocytes (Figure 5A). The mRNA expression level of tyrosinase significantly decreased in cells which were treated with 1 mM Rhododendrol, compared with control cells, and 6-SG pretreatment significantly blocked the Rhododendrol-induced decrease in tyrosinase expression (Figure 5B). 

## 3. Discussion

In this study, we demonstrated that 6-SG attenuated H_2_O_2_-induced cell damage in human epidermal melanocytes by activating the Nrf2-ARE pathway. This study discovered a novel protective effect of 6-SG on human melanocytes, suggesting that 6-SG may have potential for the treatment of pigmentary disorder diseases. In the current study, we used H_2_O_2_ (Figure 1, Figure 2, Figure 3 and Figure 4) and Rhododendrol (Figure 5) injury HEMn-MPs cellular models to study the effects of 6-SG on oxidative stress. We found that pretreatment with 6-SG provided protection to HEMn-MPs from H_2_O_2_ or Rhododendrol-induced damage. Consistent with previous reports, we found that H_2_O_2_ is cytotoxic to HEMn-MPs in a dose-dependent pattern (Figure S1A). The cell viability of HEMn-MPs incubated with H_2_O_2_ increased significantly after pretreatment with 6-SG. In addition, 6-SG also attenuated the H_2_O_2_-induced oxidative stress in HEMn-MPs, confirming the protective effect of 6-SG against oxidative stress in HEMn-MPs. The Nrf2-ARE pathway, including HO-1 and Nqo1, protects melanocytes and reduces cytotoxicity caused by oxidative stress [35]. In this study, 6-SG was shown to protect human melanocytes from H_2_O_2_-induced oxidative stress by activating the Nrf2-ARE pathway. 

Previous studies have shown that H_2_O_2_ is closely related to the onset, as well as the progression of vitiligo [6]. A recent study has shown that H_2_O_2_ decreases the antioxidant capability in the epidermis of vitiligo patients [36]. In vitiligo, H_2_O_2_ was reported to oxidize melanogenesis-related hormones and factors, such as epidermal ACTH, α-MSH, and β-endorphin, resulting in the loss of their functions via the promotion of pigmentation in melanocytes [37]. Furthermore, H_2_O_2_-mediated oxidation is also reported to affect calcium binding, disrupting calcium homeostasis and l-phenylalanine-uptake in the epidermis in acute vitiligo [38]. In addition, impaired Nrf2 signaling, decreased antioxidative enzyme levels, including HO-1, and increased oxidative stress have been reported in patients with vitiligo [39,40,41,42]. Recently, Nrf2, one of the most critical antioxidant enzymatic systems, and its downstream target genes were found to be up-regulated in non-lesional vitiligo skin biopsies, suggesting that a consistently higher Nrf2-dependent transcriptional activity is required for the maintenance of redox homeostasis in disease-free epidermis. Thus, the activation of antioxidative mechanisms is essential for the protection of melanocytes against oxidative stress in patients with vitiligo. 

Ginger is a popular, globally used spice and food preservative, that is also considered to be a life-promoting herb. It has been used in traditional medicine to treat various diseases [12,43]. In the last decade, research on the components of ginger has significantly increased. Among its components, 6-SG has displayed numerous pharmacological properties, including antioxidant, anti-inflammatory, anti-neuroinflammatory, anti-cathartic, anti-neoplastic, and hypotensive [12,44,45]. The present study demonstrates for the first time the antioxidant activity of 6-SG via the induction of Nrf2/ARE-mediated antioxidant enzyme HO-1 and Nqo1 pathway in cultured cells. 

## 4. Materials and Methods 

### 4.1. Reagents

Hydrogen peroxide (H_2_O_2_) was purchased commercially from WAKO (Osaka, Japan) and diluted with PBS. 6-SG was purchased commercially from Sigma (St. Louis, MO, USA) and dissolved in dimethyl sulfoxide (DMSO). Rhododendrol was obtained from Kanebo (Tokyo, Japan) and dissolved in DMSO.

### 4.2. Cell Culture

Normal human neonatal epidermal melanocytes from a moderately pigmented donor (HEMn-MP) were purchased from Invitrogen (Thermo Fisher Scientific, Carlsbad, CA, USA), and cultured in Medium 254 (M-254-500; Thermo Fisher Scientific) supplemented with 1% (*v/v*) human melanocyte growth supplement (Thermo Fisher Scientific) at 37°C in an atmosphere containing 5% (*v/v*) CO_2_. The melanocytes were used at passages 6–8. Cells were seeded into 6-well plates at a density of 5 × 10^5^ cells/well 12 h before treatment. Cells were treated with H_2_O_2_ at 0.1 mM and 0.2 mM (final concentration), 6-SG at 5 µM (final concentration), or Rhododendrol at 1 mM, 1.5 mM and 3 mM (final concentration) for certain periods prior to extraction of RNA and protein. Because oxidative stress has been well known to trigger an endogenous antioxidant defense system at the early stage (2 h–72 h) by itself [46], we evaluated the antioxidative ability of our agents at a later stage after a longer treatment time (140 h).

### 4.3. Cell Viability Assay

HEMn-MPs (1 × 10^4^ cells/well) were cultured in 96-well flat-bottom tissue culture plates. After experimental treatments, cells were washed three times with cold PBS, and cell viability was evaluated using a Cell Count Reagent SF colorimetric assay (Nacalai Tesque, Kyoto, Japan). Briefly, 10 μL of Cell Count Reagent SF was added to each well, and cells were incubated for 2 h at 37 °C. Cell viability was determined colorimetrically by measuring OD_450_ with a microplate reader (Model 550: Bio-Rad Laboratories, Hercules, CA, USA). The percentage of viable cells was calculated as follows: percentage viable cells = T/C × 100, where T is the mean OD_450_ of the treated group, and C is the absorbance of the control group.

### 4.4. Melanin Content Assay

To determine melanin content, cells were dissolved in 200 μL of 1 N NaOH for 30 min at 100ºC to solubilize the melanin, which was then quantified in cell suspensions by recording the absorbance at 405 nm as described previously [47]. Melanin content was calculated and corrected based on cell number.

### 4.5. RNA Isolation and Real-Time RT-PCR Analysis

Total RNA from cell pellets was isolated using the Maxwell^®^ 16 LEV simplyRNA Tissue Kit (Promega, Madison, WI, USA) following manufacturer’s instructions. The integrity of the RNA was verified by gel electrophoresis. Total RNA (100 ng) was reverse-transcribed into first-strand cDNA (ReverTra Ace^®^ qPCR RT Master Mix, TOYOBO, Osaka, Japan). The primers used for real-time PCR were as follows: Tyrosinase, 5′-TGACTCCAATTAGCCAGTTCCT-3′ (sense) and 5′-GACAGCATTCCTTCTCCATCAG-3′ (antisense); HO-1, 5′-CGAGCATAAATGTGACCGGC-3′ (sense) and 5′-CTCTGACAAATCCTGGGGCA-3′ (antisense); and GAPDH, 5′GACAGTCAGCCGCATCTTCT-3′ (sense) and 5′-GCGCCCAATACGACCAAATC-3′ (antisense). Each reaction was performed in triplicate.

### 4.6. Western Blot Analysis

Proteins from cell pellets were extracted, and 5 μg of extracted proteins was used for Western blotting analysis as described previously [48]. The following primary antibodies were used at a concentration of 1:1000: anti-Nrf2 (#12721, Cell Signaling Technology, Beverly, MA, USA), anti-Nqo1 (#3187, Cell Signaling Technology), anti-HO-1 (#70081, Cell Signaling Technology), and anti-GAPDH (#2118, Cell Signaling Technology). GAPDH was used as a loading control.

### 4.7. Oxidative Stress Assessment

For cultured human primary epidermal melanocytes, oxidative stress was detected by live imaging using CellROX^®^ Green Reagent (#C10444, Thermo Fisher Scientific Inc., MA, USA). Cells were treated with 5 µM CellROX^®^ Green Reagent for 30 min and then washed with PBS twice, applied with the indicated treatments, and then subjected to live cell imaging by phase contrast and confocal fluorescence microscopy (Keyence Biozero confocal microscope: Keyence Co., Osaka, Japan).

### 4.8. Statistical Analysis

The experiments were repeated at least three times. Data are presented as mean ± SD. Statistical analysis was conducted using two-way analysis of variance for interactions between variables. Unpaired Student’s *t*-test (Microsoft Excel: Microsoft Corp., Redmond, WA, USA) was used for comparisons between two groups. *p*-values < 0.05 were considered statistically significant.

## 5. Conclusions

In conclusion, our results show that 6-SG pretreatment protected melanocytes against H_2_O_2_-induced oxidative stress via activating the Nrf2 pathway and induction of Nrf2 and HO-1 expression. This study improves our understanding of the molecular mechanism of the beneficial effect of ginger in the treatment of vitiligo patients. These findings demonstrate that 6-SG has potential for use as an antioxidant agent in the prevention and treatment of vitiligo or other oxidative stress-associated pigmentary disorders.

## Supplementary Materials

Supplementary Materials can be found at https://www.mdpi.com/1422-0067/21/10/3537/s1.
