# Supplementary material for: 6-Shogaol Protects Human Melanocytes against Oxidative Stress through Activation of the Nrf2-Antioxidant Response Element Signaling Pathway
Source: Int J Mol Sci. 2020 May 16;21(10):3537. doi: 10.3390/ijms21103537 (PMC7279012; doi:10.3390/ijms21103537)
Supplement: Supplementary file 1 [file ijms-21-03537-s001.zip › ijms-777158-supplementary/Figure S1.docx]

**
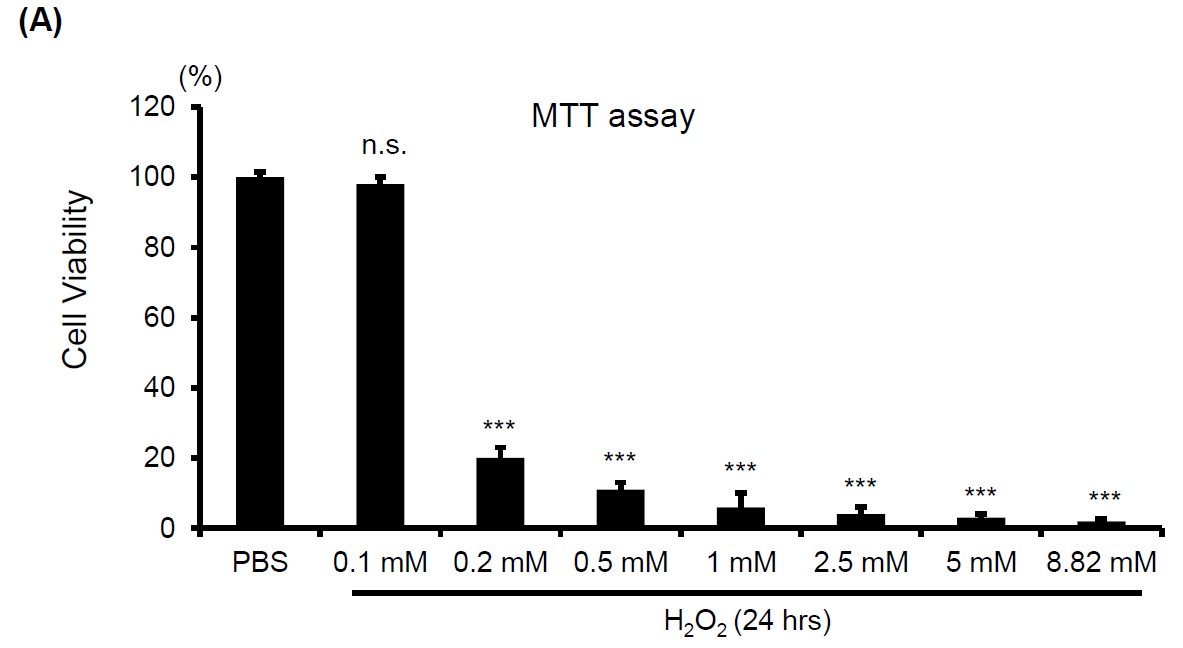
**

**
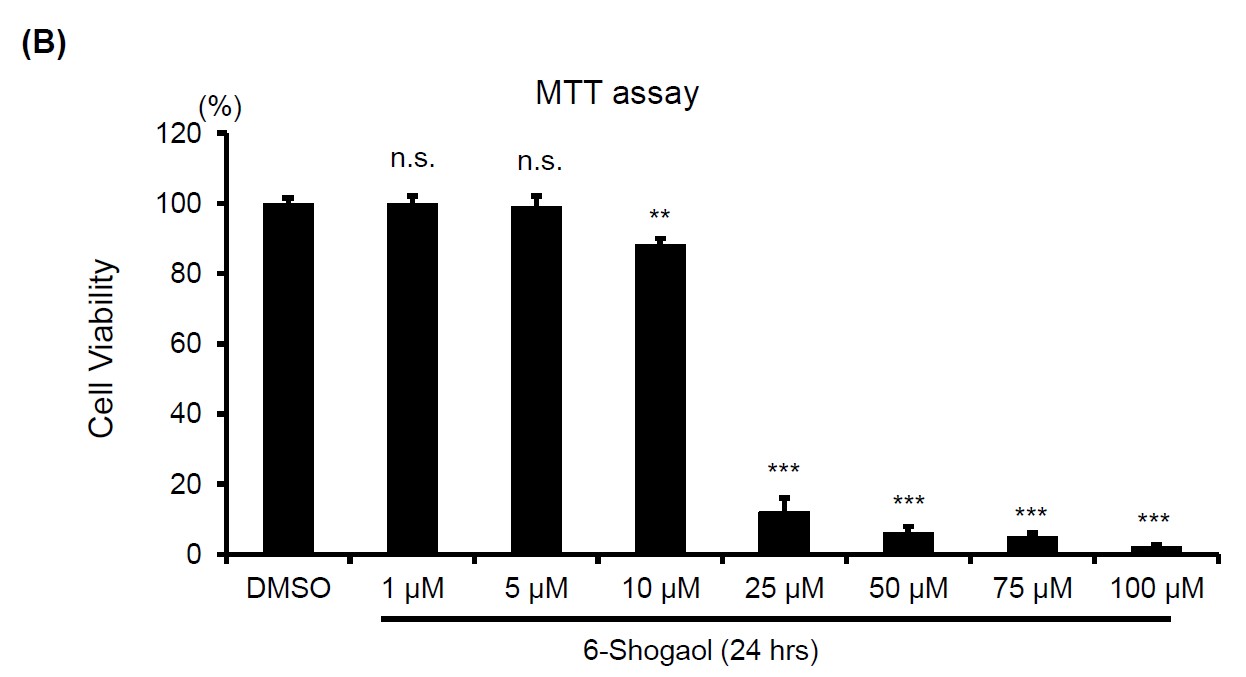
**

**Figure S1.** Cell viability determined by MTT assay. Cell viability of cultured human primary epidermal melanocytes was evaluated after exposure to the indicated increasing concentrations of H_2_O_2_ (**A**) and 6-shogaol (**B**) for 24 h. Data represent the results of three independent experiments. Data are shown as mean ± SD. n.s., no significant difference versus control; **, 0.05 < *p* < 0.01 versus control; ***, *p*< 0.01 versus control.
