# Supplementary material for: 6-Shogaol Protects Human Melanocytes against Oxidative Stress through Activation of the Nrf2-Antioxidant Response Element Signaling Pathway
Source: Int J Mol Sci. 2020 May 16;21(10):3537. doi: 10.3390/ijms21103537 (PMC7279012; doi:10.3390/ijms21103537)
Supplement: Supplementary file 1 [file ijms-21-03537-s001.zip › ijms-777158-supplementary/Legend of Video S1 and Video S2.docx]

**Video S1.** Oxidative stress live imaging in HEMn-MPs was detected using CellROX® Green reagent during exposure to 0.2 mM H2O2 for the first 12 h.

**Video S2.** Oxidative stress live imaging in 5 µM 6-SG 6h-pretreated HEMn-MPs was detected using CellROX® Green reagent during exposure to 0.2 mM H2O2 for the first 12 h.
